# Does Dexmedetomidine as a Neuraxial Adjuvant Facilitate Better Anesthesia and Analgesia? A Systematic Review and Meta-Analysis

**DOI:** 10.1371/journal.pone.0093114

**Published:** 2014-03-26

**Authors:** Huang-Hui Wu, Hong-Tao Wang, Jun-Jie Jin, Guang-Bin Cui, Ke-Cheng Zhou, Yu Chen, Guo-Zhong Chen, Yu-Lin Dong, Wen Wang

**Affiliations:** 1 Department of Anesthesiology, Fuzhou General Hospital of Nanjing Military Region, Fuzhou, PR China; 2 Unit for Evidence Based Medicine, Department of Anatomy, Histology and Embryology & K.K. Leung Brain Research Centre, Preclinical School of Medicine, Fourth Military Medical University, Xi'an, PR China; 3 Department of Burn and Cutaneous Surgery, Xi’jing Hospital, Fourth Military Medical University, Xi'an, PR China; 4 Department of Neurosurgery, Fuzhou General Hospital of Nanjing Military Region, Fuzhou, PR China; 5 Department of Diagnostic Radiology, Tangdu Hospital, Fourth Military Medical University, Xi’an, PR China; 6 China Pharmaceutical University, Nanjing, PR China; The James Cook University Hospital, United Kingdom

## Abstract

**Background:**

Neuraxial application of dexmedetomidine (DEX) as adjuvant analgesic has been invetigated in some randomized controlled trials (RCTs) but not been approved because of the inconsistency of efficacy and safety in these RCTs. We performed this meta-analysis to access the efficacy and safety of neuraxial DEX as local anaesthetic (LA) adjuvant.

**Methods:**

We searched PubMed, PsycINFO, Scopus, EMBASE, and CENTRAL databases from inception to June 2013 for RCTs that investigated the analgesia efficacy and safety for neuraxial application DEX as LA adjuvant. Effects were summarized using standardized mean differences (SMDs), weighed mean differences (WMDs) or odds ratio (OR) with suitable effect model. The primary outcomes were postoperative pain intensity and analgesic duration, bradycardia and hypotension.

**Results:**

Sixteen RCTs involving 1092 participants were included. Neuraxial DEX significantly decreased postoperative pain intensity (SMD, −1.29; 95% confidence interval (CI), −1.70 to −0.89; *P*<0.00001), prolonged analgesic duration (WMD, 6.93 hours; 95% CI, 5.23 to 8.62; *P*<0.00001) and increased the risk of bradycardia (OR, 2.68; 95% CI, 1.18 to 6.10; *P* = 0.02). No evidence showed that neuraxial DEX increased the risk of other adverse events, such as hypotension (OR, 1.54; 95% CI, 0.83 to 2.85; *P* = 0.17). Additionally, neuraxial DEX was associated with beneficial alterations in postoperative sedation scores and number of analgesic requirements, sensory and motor block characteristics, and intro-operative hemodynamics.

**Conclusion:**

Neuraxial DEX is a favorable LA adjuvant with better and longer analgesia. The greatest concern is bradycardia. Further large sample trials with strict design and focusing on long-term outcomes are needed.

## Introduction

Neuraxial anesthesia and analgesia provide solid analgesic effect by inhibiting nociceptive transmission from peripheral to central neuronal system [1], [2]. However, their analgesic advantages might be limited by the short life of current local anesthetics (LAs), and, especially, be weakened during postoperative pain control [3]. The analgesic duration can be prolonged by increasing dose of LA, however, the risk of accompanied systemic and potential neurotoxicity can also be increased. Therefore, adjunct analgesic strategy is an alternative to prolong the analgesic duration, decrease the potential risk of side effects by reducing the dose of individual LA. Recently, several neuraxial adjuvants, including clonidine [4], opioids [5]–[7], dexamethasone [8], ketamine [9], magnesium [10], and midazolam [11] have demonstrated the synergistic analgesic effect with LAs with varying degrees of success.

Dexmedetomidine (DEX) is a clinically used anesthetic and belongs to high selective α_2_-adrenergic receptors (α_2_AR) agonist. Intravenous DEX exhibits synergism with regional anesthesia and facilitates postoperative pain control [12], [13] and has been accepted as a clinical anesthetic strategy. However, DEX has not been approved by US Food and Drug Administration (FDA) for neuraxial administration. Pre-clinic evidences showed that neuraxial DEX produces antinociception by inhibiting the activation of spinal microglia and astrocyte [14], [15], decreasing noxious stimuli evoked release of nociceptive substances [16], and further interrupting the spinal neuron-glia cross talk and regulating the nociceptive transmission under chronic pain condition [17]. Thus, DEX might be an interesting adjuvant for neuraxial anesthesia and analgesia to decrease intra- and postoperative anesthetic consumption and prolong the postoperative analgesic duration, but the potentially increased risk of bradycardia, hypotension and neurotoxicity should be taken into consideration in clinic settings. One recent meta-analysis reported the facilitatory effects of perineural DEX on neuraxial and peripheral nerve block [18] and another suggested beneficial effects of intravenous and intrathecal DEX in spinal anesthesia [19]. However, the results from these two meta-analyses might be biased, because 1. the pooled results were not based on all the currently available RCTs on neuraxial DEX; 2. only the primary outcomes of the sensory and motor block durations were pooled for neuraxial DEX; 3. the analgesic and side effects of adjunct neuraxial DEX to LA has not been carefully investigated; 4. no effort was made to explore the significant heterogeneity within the RCTs. Thus, we performed the current systematic review and meta-analysis focusing on postoperative pain outcomes (pain intensity and analgesic duration) and major adverse events (bradycardia and hypotension) of neuraxial DEX as an adjuvant compared with LA alone.

## Methods

We performed the current meta-analysis based on the QUORUM (Quality of Reporting of Meta-analyses) guidelines [20] and the recommendations of the Cochrane Collaboration [21].

### Literature Search

The electronic databases screened were MEDLINE (1990 to June 2013), PsycINFO (1990 to June 2013), Scopus (1988 to June 2013), EMBASE (1990 to June 2013), and the Cochrane Library (Issue 6 of 12, June 2013), including the Cochrane Central Register of Controlled Trials (CENTRAL), Cochrane Database of Systematic Reviews (CDSR), Database of Abstracts of Reviews of Effects (DARE), and Health Technology Assessments (HTA) using the search phrases (intrathecal OR spinal OR subarachnoid OR epidural OR caudal OR intravertebral OR neuraxial) AND (dexmedetomidine OR DEX OR Precedex). Filters were used in PubMed and EMBase to exclude animal studies. A hand search in reference sections of included trials, published meta-analyses, and relevant review articles was conducted to identify additional articles.

### Study Selection

Selected studies met the following criteria: 1. Any randomized controlled trial (RCT), controlled clinical trial, or open label trial (OLT) designed with at least two groups that one control group receiving pharmacological placebo (saline) in combination with one LA, and the other group receiving DEX in combination with one LA; 2. Neuraxial DEX was delivered via any intravertebral routes, such as epidural, intrathecal, and caudal route in adults and children of any sex undergoing selective surgical procedural; 3. Trials revealed at least one of primary or secondary outcomes mentioned below.

### Outcome Measurement

Primary outcomes were postoperative pain intensity within 24 hours, postoperative analgesia duration (“time to first analgesic requirement” in hours), and major adverse events, including bradycardia and hypotension. Postoperative pain scores from trials measured by verbal rating scale (VRS), visual analog scale (VAS), pediatric observational face, leg, activity, cry, consolability (FLACC) pain scale, or children's and infant's post-operative pain scale (CHIPPS) were pooled to evaluate postoperative pain intensity. Four postoperative time points were pooled to assess pain, 2 to 4 h, 6 to 8 h, 12 h, and 24 h. Different dosages of DEX from the included studies were pooled and the dose effect was not stratified in the current study.

Secondary outcomes were the number of postoperative analgesic requirements, postoperative sedation scores (2 to 4 h, 6 to 8 h, 12 h, and 24 h measured by 2-point scale, 4-point scale, or 5-point scale), sensory and motor block characteristics (onset and duration), intra-operative hemodynamic (heart rate (HR) and mean arterial pressure (MAP) measured on 0∼30 min, 30∼60 min, and > 60 min), and other adverse events (nausea, vomiting, itching, respiratory depression, urinary retention, additional sedation, shivering, hypoxemia, cardiac arrhythmia, and agitation).

### Data Extraction

Characteristics of patients (number of patients, American Society of Anesthesiologist (ASA) rating, age, gender, type of surgery and anesthesia, body mass index (BMI)) and trials design (intervention, follow-up time, completed rate and reported outcomes) were also recorded. If the data mentioned above were unavailable in the article, the corresponding authors were contacted for missing information. If the outcomes in the published studies were presented in a graph manner without any description of absolute value, Image J software Version 2.1.4.7 (Image J software, National Institutes of Health, USA, http://imagej.nih.gov) was used to restore the related data if we could not get the original data from the authors.

All data were independently extracted using a standard data collection forms by 2 reviewers (HH Wu and HT Wang), and then the collected data were checked and entered into Review Manager analyses software (RevMan) Version 5.2.7 using the double-entry system by the other 2 reviewers (JJ Jin and GB Cui). All discrepancies were rechecked and consensus was reached by discussion with a third author (KC Zhou) involved. A record of reasons for excluding studies was kept. Cohen's kappa was applied for calculating inter-rater agreement.

### Assessment of Study Quality

A critical evaluation of the included studies quality was performed by 2 reviewers (KC Zhou and Y Chen) by using a 5-point Jadad scale [22]. The main categories consisted of the following 5 items: “Was the study described as randomized? (1)”, “Was the method used to generate the sequence of randomization described and appropriate (random numbers, computer-generated, etc)? (1)”, “Was the study described as double-blind? (1)”, “Was the method of double-blinding described and appropriate (identical placebo, active placebo, dummy, etc)? (1)”, and “Was there a description of withdrawals and drop-outs? (1)”. A score of 4 to 5 was considered a high methodological quality.

### Assessment of Risk of Bias

Two reviewers (JJ Jin and GB Cui) independently evaluated the risk of bias according to the recommendations from the Cochrane collaboration [19], [23]. The main categories consisted of random sequence generation, allocation concealment, blinding of participants and personnel, blinding of outcome assessment, incomplete outcome data, and selective reporting. Each domain was assessed to “high risk”, “low risk”, or “unclear”.

### Assessment of Heterogeneity and Publication Bias

We pooled all studies reporting the same primary or secondary outcomes together. And then the study heterogeneity at overall level was investigated by using a χ^2^ test and calculating I^2^ statistic [19], [24]. When I^2^ was 50% or lower, a low heterogeneity was rated and the data were pooled with a fixed effect model. When I^2^ was over 50%, a significant heterogeneity was rated and the data were pooled with a random effects model [24].

Subgroup analyses were used to identify the significant heterogeneity according to different routes of DEX delivery (epidural, intrathecal, and caudal route), different doses of DEX (≤ 5 μg and > 5 μg), and different time points after DEX administration (2∼4 h, 6∼8 h, 12 h, and 24 h or ≤ 60 min and > 60 min). Furthermore, meta-regression was used to identify the origin of heterogeneity, such as the different routes, doses, time points, and study qualities.

We performed the sensitivity analyses to examine the effect of primary outcomes by excluding studies with low quality or high risk of bias, and investigated the potential publication bias by using graphical (Begg’s funnel plot) [25] and statistical tests (Egger’s test) [26].

### Statistical Analysis

Continuous variables were pooled by using either the standardized (SMD) or weighted mean difference (WMD) with their 95% confidence intervals (CIs). If the 95% CI covered the value of 0, we considered that the difference between DEX and placebo group was not statistically significant. SMD was calculated for postoperative pain intensity and postoperative sedation scores, because they were measured with different scales. WMD was calculated for postoperative analgesia duration, sensory and motor block characteristics, and intra-operative hemodynamic, because they were measured by the same scale. If these continuous data were only reported as mean or median with standard error or range, we converted them into mean with standard deviation (SD) as previously reported [27]. Binary variables (the number of postoperative analgesic requirements, and the primary and secondary adverse events) were pooled by using odds ratio (OR) with 95% CIs. If the 95% CI covered the value of 1, we considered that the difference between DEX and placebo group was not statistically significant. For the number of postoperative analgesic requirements and the adverse events with statistically significant difference between the DEX and placebo group, number need to treat (NNT) or number need to harm (NNH) was further calculated. The meta-analyses were performed with RevMan 5.2.7 according to Cochrane Handbook for Systematic Reviews of Interventions [24] and further confirmed by using Stata 12.0 software (Stata Corporation, USA).

## Results

### Search Results

**Figure 1:**
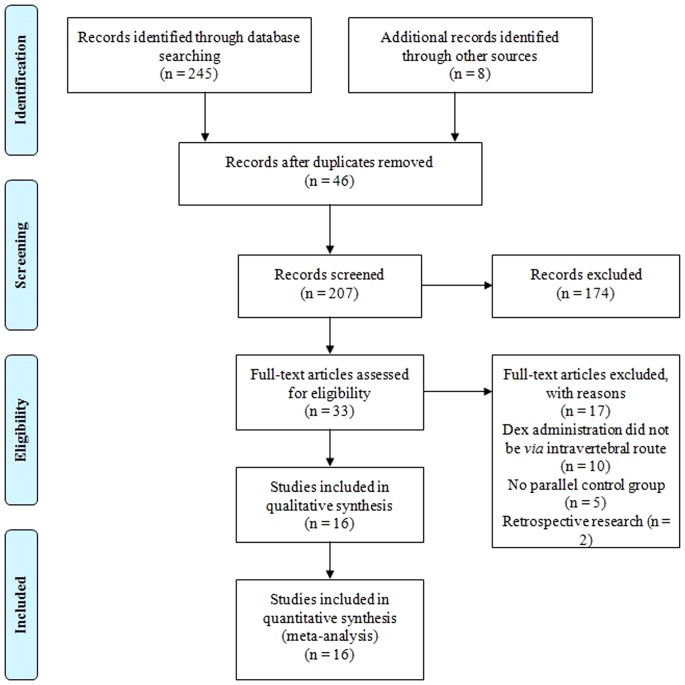
PRISMA flow diagram of search strategy and study selection.

The literature search yielded 253 citations. Initially, 46 records were removed because of duplicate publication. On a more detailed review, an additional 174 papers were excluded for the following reasons: pre-clinical experiments, comments, editorial, case reports, reviews, and data unavailable. Seventeen more papers were further excluded because of DEX *via* systemic or nasal route, lacking of parallel placebo control, and retrospective research. Finally, the remained 16 studies [28]–[43] with available data met our selection criteria and were included in the meta-analysis. The flow diagram of search strategy and study selection was presented in Figure 1.

### Characteristics of the Included Studies

All 16 included studies [28]–[43] were designed as prospective, randomized, double-blinded and placebo controlled trials, and their main characteristics were presented in Table S1. Patients investigated in 4 trials [29], [31], [35], [41] were children, in 1 trial [38] were full term parturients, and in 11 trials [28], [30], [32]–[34], [36], [37], [39], [40], [42], were adults of any sex. DEX delivery *via* epidural route was reported in 4 trials [30], , *via* intrathecal route was reported in 8 trials [28], [32]–[34], [39], [40], [42], , and *via* caudal route was reported in 4 trials [29], [31], [35], [41]. The doses of DEX varied from 1 to 2 μg/kg *via* epidural and caudal route, and 3 to 15 μg *via* intrathecal route. In total, 470 patients were randomly assigned to receive neuraxial administration of DEX combined with bupivacaine or ropivacaine, and 401 patients were assigned to placebo groups receiving neuraxial administration of saline and bupivacaine or ropivacaine.

### Methodological Quality and Risk of Bias

The Jadad score of each included study was presented in Table S1, and the median quality score was 4 (range from 3 to 5). Inter-rater reliability for this assessment was κ = 0.79.

The risk of bias of included studies was presented in Table S2. In total, 8 trials (50%) [28], [29], [31], [35], [39], [40], [42], [43] clearly described the procedure of randomization, and 9 trials (56%) [28], [30], [31], [35], [38]–[40], [42], reporting the methods of allocation concealment. All trials [28]–[43] were double-blinded for participants and personnel as well as outcome assessment. One trial (6%) [38] was rated with high risk of attribution bias. All trials [28]–[43] had an unclear risk of bias on selective outcome reporting. Nine trials (56%) [28]–[31], [35], [39], [40], [42], had a low risk of bias on other sources of bias.

### Meta-Analyses of Primary Outcomes

#### Postoperative pain intensity

**Figure 2:**
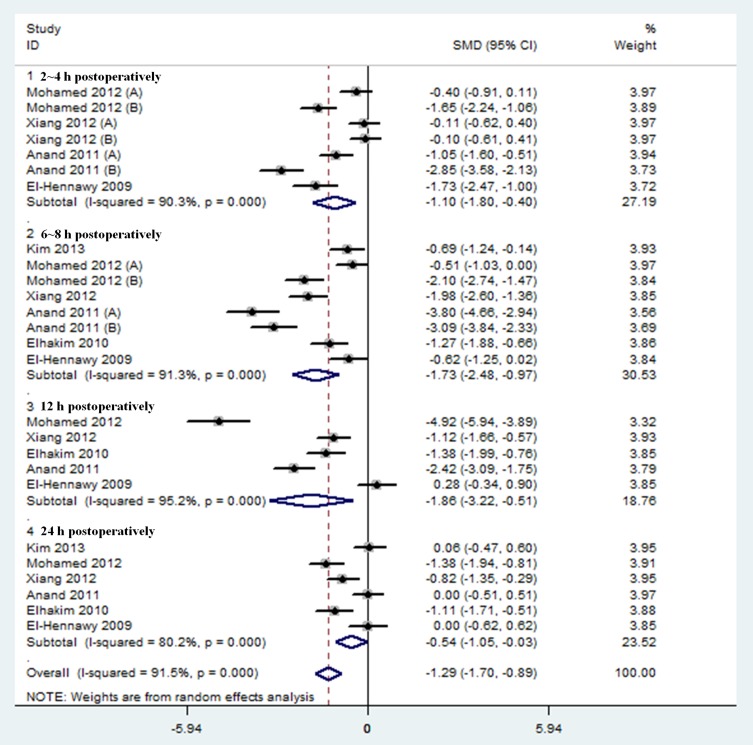
Forest plot: Postoperative pain intensity within 24 hours.

Results were presented in Figure 2 and Table 1. Postoperative pain intensity within 24 hours was investigated in 6 trials [29]–[31], [34], [41], . The pooled analysis revealed that neuraxial DEX was associated with a significant reduction of postoperative pain intensity within 24 hours compared with placebo group (SMD, −1.29; 95% CI, −1.70 to −0.89; *P* < 0.00001). The I^2^ value of 92% indicated significant heterogeneity.

**Table 1:** Effect sizes and subgroup analysis of DEX on postoperative pain intensity and duration.

Subgroup	Postoperative pain intensity				Postoperative analgesia duration (h)			
	N*	Effect size^#^ (95% CI)	I^2^ test (%)	*P* value	N*	Effect size^#^ (95% CI)	I^2^ test (%)	*P* value
All studies	6 (324)	−1.29 (−1.70, −0.89)	92	< 0.00001	8 (440)	6.93 (5.23, 8.62)	98	< 0.00001
**DEX route**								
DEX *via* epidural route	1 (50)	−1.23 (−1.58, −0.88)	0	< 0.00001	1 (36)	2.00 (0.65, 3.35)	NA	0.004
DEX *via* intrathecal route	2 (114)	−1.36 (−2.13, −0.59)	93	0.0005	4 (234)	4.27 (3.27, 5.26)	93	< 0.00001
DEX *via* caudal route	3 (160)	−1.01 (−1.53, −0.50)	92	0.0001	3 (170)	10.70 (8.45, 12.95)	88	< 0.00001
**DEX dose**								
≤5 μg	2 (114)	−1.36 (−2.13, −0.59)	93	0.0005	4 (234)	4.27 (3.27, 5.26)	93	< 0.00001
> 5 μg	4 (210)	−1.04 (−1.49, −0.59)	91	< 0.00001	4 (206)	8.56 (4.42, 12.70)	98	< 0.0001
**Postoperative time**								
2∼4 h	4 (220)	−1.10 (−1.80, −0.40)	91	0.002	—	—	—	—
6∼8 h	6 (324)	−1.73 (−2.48, −0.97)	92	< 0.00001	—	—	—	—
12 h	5 (270)	−1.86 (−3.22, −0.51)	96	0.007	—	—	—	—
24 h	6 (324)	−0.54 (−1.05, −0.03)	81	0.04	—	—	—	—

* Number of trials pooled (total number of subjects).

# Effect size: standardized mean difference for postoperative pain intensity and weighted mean difference for postoperative analgesia duration (h).

Further subgroup analyses according to different routes and doses of neuraxial DEX, as well as time periods during postoperative care did not affect the pooled results, and all of these analyses were also influenced by heterogeneity.

#### Postoperative analgesia duration (“time to first analgesic requirement” in hours)

**Figure 3:**
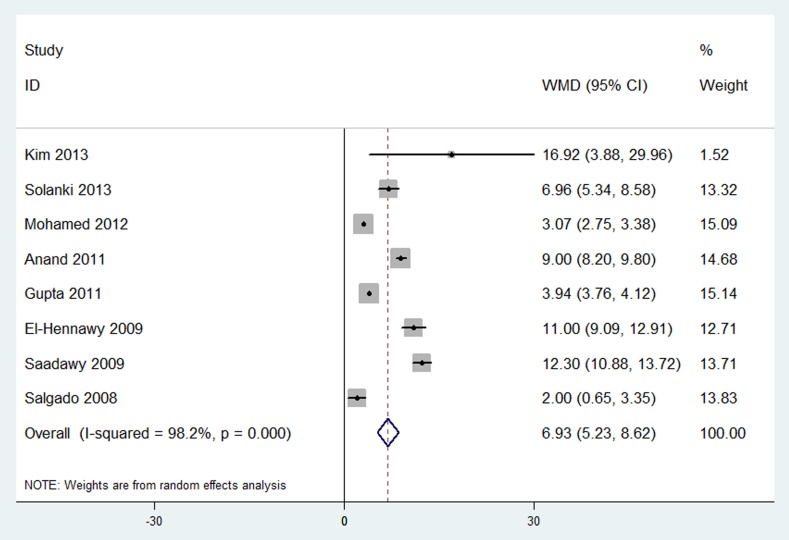
Forest plot: Postoperative analgesic duration.

Results were presented in Figure 3 and Table 1. Postoperative analgesia duration was investigated in 8 trials [29], [31], [32], [34]–[36], [40], . The pooled analysis revealed that neuraxial DEX was associated with a significantly prolonged analgesia duration compared with placebo group (WMD, 6.93 hours; 95% CI, 5.23 to 8.62; *P* < 0.00001). The I^2^ value of 98% indicated significant heterogeneity.

Further subgroup analyses according to different routes and doses of neuraxial DEX did not affect the pooled results, and all of these analyses were also influenced by heterogeneity.

#### Bradycardia

**Figure 4:**
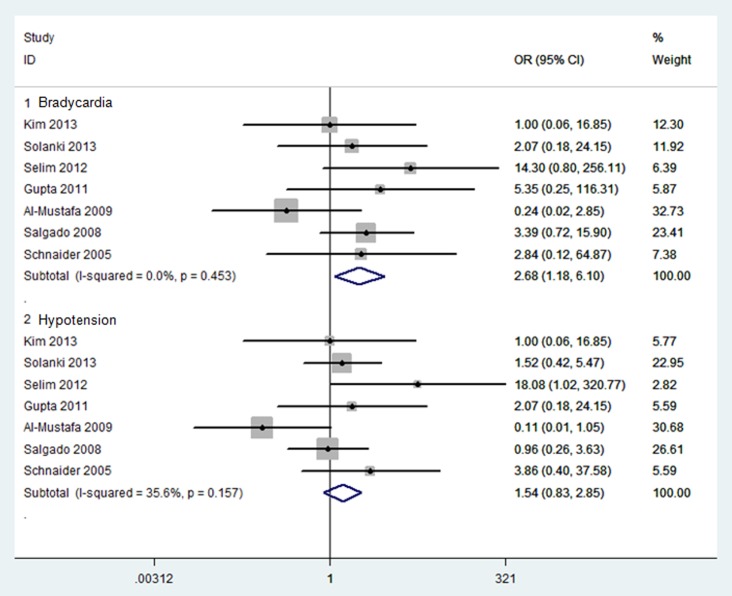
Forest plot: Primary adverse events: bradycardia and hypotension.

Results were presented in Figure 4. Bradycardia was investigated in 7 trials [28], [32], [36]–[38], [40], . The pooled analysis revealed that neuraxial DEX was associated with a significantly higher incidence of bradycardia compared with placebo group (OR, 2.68; 95% CI, 1.18 to 6.10; *P* = 0.02; NNH = 14). Bradycardia outcome showed no any heterogeneity (I^2^ = 0%).

#### Hypotension

Results were presented in Figure 4. Hypotension was investigated in 7 trials [28], [32], [36]–[38], [40], with no difference between neuraxial DEX and placebo group (OR, 1.54; 95% CI, 0.83 to 2.85; *P* = 0.17). Hypotension outcome showed less heterogeneity (I^2^ = 36%).

### Meta-Analyses of Secondary Outcomes

#### The number of postoperative analgesic requirements

**Figure 5:**
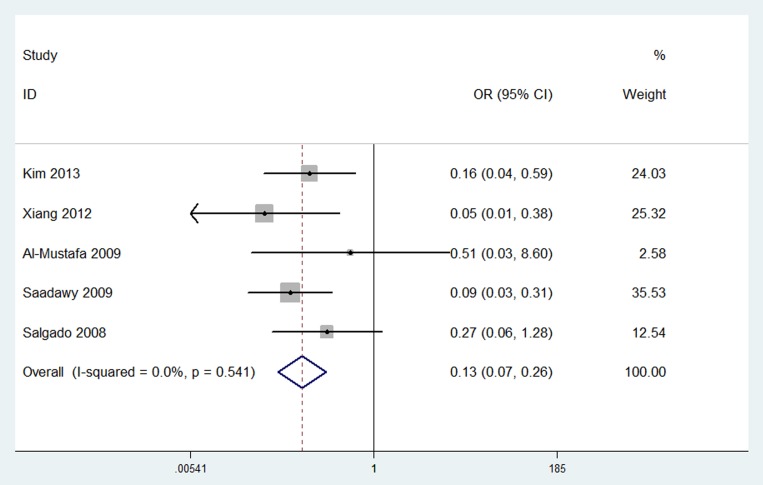
Forest plot: The number of postoperative analgesic requirements.

Results were presented in Figure 5. The number of postoperative analgesic requirements was investigated in 5 trials [28], [35], [36], [41], [42]. The pooled analysis revealed that neuraxial DEX was associated with a significant reduction in the number of postoperative analgesic requirements compared with placebo group (OR, 0.13; 95% CI, 0.07 to 0.26; *P* < 0.00001) without any heterogeneity (I^2^ = 0%).

#### Postoperative sedation scores

Results were presented in Table 2. Postoperative sedation scores within 24 hours were investigated in 3 trials [30], [35], [41]. The pooled analysis revealed that neuraxial DEX was associated with a significant increase of postoperative sedation within 24 hours compared with placebo group (SMD, 0.96; 95% CI, 0.16 to 1.76; *P* = 0.02). The I^2^ value of 94% indicated significant heterogeneity.

**Table 2:** Effect sizes and subgroup analysis of DEX on postoperative sedation.

Subgroup	N*	Effect size^#^ (95% CI)	I^2^ test (%)	*P* value
**All studies**	3 (170)	0.94 (0.16, 1.76)	94	0.02
**DEX route**				
DEX *via* epidural route	1 (50)	0.26 (−0.19, 0.71)	0	0.25
DEX *via* caudal route	2 (120)	0.64 (0.43, 0.86)	86	< 0.00001
**Postoperative time**				
2∼4 h	2 (120)	0.61 (−1.98, 3.20)	98	0.64
6∼8 h	3 (170)	1.01 (−0.58, 2.59)	95	0.21
12 h	2 (110)	0.68 (−0.32, 1.67)	84	0.18
24 h	2 (110)	1.03 (0.34, 1.72)	97	0.30

* Number of trials pooled (total number of subjects).

# Effect size: standardized mean difference for postoperative sedation (point).

Further subgroup analysis investigating different routes of neuraxial DEX revealed that there was no difference between epidural DEX and placebo group in postoperative sedation scores. In contrast, a significantly increased postoperative sedation level was associated with caudal DEX. Another subgroup analysis investigating different time periods showed that there was no difference between neuraxial DEX and placebo group in postoperative sedation scores, and all of these analyses were also influenced by heterogeneity.

#### Sensory block characteristics

Results were presented in Table 3. The onset and duration of sensory block were investigated in 7 [28], [32], [36], [39], [40], [42], [43] and 6 trials [32], [33], [39], [40], [42], [43], respectively. The pooled analysis revealed that neuraxial DEX was associated with a significantly quick onset (WMD, −1.49 minutes; 95% CI, −2.28 to −0.70; *P* = 0.0002) and prolonged duration (WMD, 101.15 minutes; 95% CI, 58.09 to 144.22; *P* < 0.00001) of sensory block compared with placebo group. The I^2^ value of 89% and 98% indicated significant heterogeneity both in onset and duration of sensory block, respectively.

**Table 3:** Effect sizes and subgroup analysis of DEX on sensory block characteristics.

Subgroup	Onset of sensory block (min)				Duration of sensory block (min)			
	N*	Effect size^#^ (95% CI)	I^2^ test (%)	*P* value	N*	Effect size^#^ (95% CI)	I^2^ test (%)	*P* value
**All studies**	7 (381)	−1.49 (−2.28, −0.70)	89	0.0002	6 (316)	101.15 (58.09, 144.22)	98	< 0.00001
**DEX route**								
DEX *via* epidural route	1 (36)	−0.40 (−1.21, 0.41)	NA	0.33	—	—	—	—
DEX *via* intrathecal route	6 (345)	−1.67 (−2.57, −0.77)	90	0.0003	6 (316)	101.15 (58.09, 144.22)	98	< 0.00001
**DEX dose**								
≤5 μg	4 (217)	−1.09 (−2.03, −0.16)	85	0.02	4 (209)	43.06 (23.86, 62.26)	88	< 0.0001
> 5 μg	4 (186)	−1.78 (−3.14, −0.41)	85	0.01	2 (107)	102.33 (24.3, 180.34)	97	0.01
**Regression dermatomes**								
2 dermatomes	—	—	—	—	6 (316)	67.22 (39.73, 94.72)	96	< 0.00001
> 2 dermatomes	—	—	—	—	4 (202)	135.39 (59.90, 210.89)	97	0.0004

* Number of trials pooled (total number of subjects).

# Effect size: weighted mean difference for both onset and duration of sensory block (min).

**Abbreviations: NA,** not applicable.

Further subgroup analysis investigating different routes of neuraxial DEX revealed that there was no difference between epidural DEX and placebo group in onset of sensory block. In contrast, a significantly fast onset of sensory block was associated with intrathecal DEX. Another subgroup analysis investigating different doses did not affect the pooled results, and all of these analyses were also influenced by heterogeneity.

All subgroup analyses investigating different routes and doses of neuraxial DEX, as well as regression dermatomes of sensory block did not affect the pooled results in duration of sensory block, and all of these analyses were also influenced by heterogeneity.

#### Motor block characteristics

Results were presented in Table 4. The onset and duration of motor block were investigated in 3 [28], [39], [40] and 7 trials [28], [33], [35], [36], [39], [40], [43], respectively. The pooled analysis revealed no difference between neuraxial DEX and placebo group in onset of motor block (WMD, −3.26 minutes; 95% CI, −6.35 to 0.02; *P* = 0.05), and a significantly prolonged duration of motor block with neuraxial DEX (WMD, 103.37 minutes; 95% CI, 57.03 to 149.71; *P* < 0.0001). The I^2^ value of 95% and 97% indicated significant heterogeneity both in onset and duration of motor block, respectively.

**Table 4:** Effect sizes and subgroup analysis of DEX on motor block characteristics.

Subgroup	Onset of motor block (min)				Duration of motor block (min)			
	N*	Effect size^#^ (95% CI)	I^2^ test (%)	*P* value	N*	Effect size^#^ (95% CI)	I^2^ test (%)	*P* value
**All studies**	3 (163)	−3.26 (−6.35, 0.02)	95	0.05	7 (341)	103.37 (57.03, 149.71)	97	< 0.0001
**DEX route**								
DEX *via* epidural route	—	—	—	—	1 (36)	90.00 (36.47, 143.53)	NA	0.001
DEX *via* intrathecal route	3 (163)	−3.26 (−6.35, 0.02)	95	0.05	5 (245)	119.71 (82.80, 156.62)	94	< 0.00001
DEX *via* caudal route	—	—	—	—	1 (60)	8.00 (−9.05, 25.05)	NA	0.36
**DEX dose**								
≤5 μg	2 (103)	−2.40 (−7.50, 2.70)	92	0.36	3 (138)	90.51 (66.11, 114.92)	56	< 0.00001
> 5 μg	2 (103)	−4.15 (−10.77, 2.46)	98	0.22	5 (246)	110.87 (40.58, 181.17)	98	0.002

* Number of trials pooled (total number of subjects).

# Effect size: weighted mean difference for both onset and duration of motor block (min).

**Abbreviations: NA,** not applicable.

Further all subgroup analyses investigating different routes and doses of neuraxial DEX did not affect the pooled results of onset of motor block.

Further subgroup analysis investigating different routes of neuraxial DEX revealed that there was no difference between caudal DEX and placebo group in duration of motor block. In contrast, a significantly prolonged duration of motor block was associated with both epidural DEX and intrathecal DEX. Another subgroup analysis investigating different doses did not affect the pooled results, and all of these analyses were also influenced by heterogeneity.

#### Intra-operative hemodynamic

Results were presented in Table 5. The intra-operative HR and MAP were investigated in 7 [28], [29], [31], [33]–[35], and 6 trials [28], [29], [31], [33], [35], [42], respectively. The pooled analysis revealed that neuraxial DEX was associated with a significantly increased HR (WMD, 1.39 bpm; 95% CI, 0.29 to 2.49; *P* = 0.01) and decreased MAP (WMD, −1.93 mmHg; 95% CI, −3.23 to −0.64; *P* = 0.004) compared with placebo group. The I^2^ value of 94% and 68% indicated significant heterogeneity both in HR and MAP, respectively.

**Table 5:** Effect sizes and subgroup analysis of DEX on hemodynamic.

Subgroup	HR (bpm)				MAP (mmHg)			
	N*	Effect size^#^ (95% CI)	I^2^ test (%)	*P* value	N*	Effect size^#^ (95% CI)	I^2^ test (%)	*P* value
All studies	7 (373)	1.39 (0.29, 2.49)	94	0.01	6 (313)	−1.93 (−3.23, −0.64)	68	0.004
Time after DEX administration								
≤ 60 min	7 (373)	2.00 (0.63, 3.38)	95	0.004	6 (313)	−1.39 (−2.75, −0.04)	69	0.04
> 60 min	3 (160)	−2.01 (−3.53, −0.49)	80	0.009	2 (89)	−6.43 (−9.21, −3.65)	0	< 0.00001
DEX route								
DEX *via* intrathecal route	4 (213)	1.56 (0.42, 2.70)	94	0.007	3 (153)	−1.97 (−3.40, −0.54)	68	0.007
DEX *via* caudal route	3 (160)	−0.63 (−3.06, 1.81)	0	0.61	3 (160)	−1.64 (−5.22, 1.95)	81	0.37
DEX dose								
≤ 5 μg	4 (192)	0.37 (−0.75, 1.49)	94	0.52	3 (132)	−2.49 (−4.10, −0.88)	61	0.002
> 5 μg	4 (203)	4.42 (2.03, 6.81)	71	0.0003	4 (203)	−0.92 (−3.22, 1.37)	79	0.43

* Number of trials pooled (total number of subjects).

# Effect size: weighted mean difference for both HR (bpm) and MAP (mmHg).

**Abbreviations: MAP**, mean arterial pressure; **HR**, heart rate.

Further subgroup analysis investigating different time periods of neuraxial DEX revealed that a significantly increased HR and decreased MAP were associated with neuraxial DEX within 60 minutes. In contrast, a significantly decreased HR and MAP were associated with neuraxial DEX beyond 60 minutes. Subgroup analysis investigating different routes of neuraxial DEX revealed that a significantly increased HR and decreased MAP were associated with intrathecal DEX. In contrast, no difference between caudal DEX and placebo group was detected in both HR and MAP. And subgroup analysis investigating different doses of neuraxial DEX revealed that a slight change of HR and significantly decreased MAP were associated with small dose of DEX (≤ 5 μg). In contrast, a significantly increased HR and slight change of MAP were associated with high dose of DEX (> 5 μg).

#### Secondary adverse events

Results were presented in Table 6. Thirteen studies [28], [31]–[38], reported the secondary adverse events including nausea [32]–[34], [36], [38], [40], [42], , vomiting [28], [33]–[35], [38], [41], , itching [31], [34], [38], [43], respiratory depression [32], [38], [40], [43], urinary retention [31], [35], [41], additional sedation [32], [36], [40], shivering [32], [36], [40], hypoxemia [36], [40], cardiac arrhythmia [34] and agitation [35] in a total of 701 patients. The pooled analysis revealed no group difference between neuraxial DEX and placebo group in all secondary adverse events except for additional sedation (OR, 0.15; 95% CI, 0.03 to 0.64; *P* = 0.01; NNT = 11). All of these analyses showed no heterogeneity (I^2^ = 0%).

**Table 6:** Summary of secondary adverse events.

Adverse events	No. of studies	DEX group no./total (%)	Saline group no./total (%)	Effect size^#^ (95% CI)	I^2^ test (%)	*P* value	NNH/NNT
Nausea	8	22/227 (9.69)	20/192 (10.42)	0.99 (0.51, 1.89)	0	0.97	—
Vomiting	7	9/223 (4.04)	11/170 (6.47)	0.70 (0.28, 1.76)	0	0.45	—
Itching	4	2/125 (1.60)	2/89 (2.25)	0.76 (0.12, 4.87)	0	0.77	—
Respiratory depression	4	0/135 (0)	0/99 (0)	NE	NA	NA	—
Urine retention	3	1/80 (1.25)	3/80 (3.75)	0.49 (0.09, 2.74)	0	0.41	—
Additional sedation	3	5/79 (6.33)	12/77 (15.58)	0.15 (0.03, 0.64)	NA	0.01	NNT 11.1
Shivering	3	8/79 (10.13)	6/77 (7.79)	1.28 (0.42, 3.85)	0	0.66	—
Hypoxemia	2	4/49 (8.16)	5/47 (10.64)	0.64 (0.14, 2.92)	NA	0.56	—
Cardiac arrhythmia	1	0/30 (0)	0/30 (0)	NE	NA	NA	—
Agitation	1	2/30 (6.67)	8/30 (26.67)	0.20 (0.04, 1.02)	NA	0.05	—

# Effect size: odd ratios for adverse events.

**Abbreviations: NA**, not applicable; **NE**, not estimable, **NNH**, number need to harm; **NNT**, number need to treat.

### Test of Heterogeneity

Significant heterogeneity was carefully considered in 2 primary outcomes (postoperative pain intensity and analgesia duration) and 4 secondary outcomes.

#### Subgroup analyses

Subgroup analyses were performed to identify the potential clinical heterogeneity according to different routes (epidural, intrathecal and caudal route) and doses (≤ 5 μg and > 5 μg) of neuraxial DEX, different time periods after neuraxial DEX administration (2∼4 h, 4∼6 h, 12 h, and 24 h, or ≤ 60 min and > 60 min), and different regression dermatomes of block level (2 dermatomes and > 2 dermatomes). However, the potential clinical heterogeneity failed to explain the study heterogeneity.

#### Meta-regression

Meta-regression was performed for postoperative pain intensity and analgesia duration to identify the potential sources of methodological and clinical heterogeneity. Evidence showed that neither route (*P*_adjusted_ = 0.97 for postoperative pain intensity and 0.91 for postoperative analgesia duration), dose (*P*_adjusted_ = 0.95 for postoperative pain intensity and 0.96 for postoperative analgesia duration), time period (*P*_adjusted_ = 0.41 for postoperative pain intensity), nor quality (*P*_adjusted_ = 0.28 for postoperative pain intensity and 0.71 for postoperative analgesia duration) was contributed to the study heterogeneity.

### Sensitivity Analyses

Sensitivity analyses were performed in 4 primary outcomes by excluding studies with low quality or high risk of bias. All the meta-analyses results were not affected by the low quality or high risk of bias of studies (Figure S1).

### Publication Bias

Publication bias was found in one primary outcome (postoperative pain intensity) according to both Begg’s funnel plot (Figure S2) and Egger’s test (Table S3).

## Discussion

The current systematic review and meta-analysis indicated that DEX as a neuraxial adjuvant was associated with reduction in postoperative pain intensity within 24 hours. The mean duration of postoperative analgesia was prolonged by approximate 7 hours. Additionally, neuraxial DEX was also associated with significantly quick onset of sensory block and prolonged duration of sensory and motor block. Intra-operative HR and MAP were also significantly affected by neuraxial DEX. No evidence showed that neuraxial DEX significantly increased the risk of drug-related adverse events, such as hypotension, nausea and vomiting, except for bradycardia.

Several reviews highlight the potential role of α_2_AR agonists for postoperative pain control [44–[46]. DEX, with its more favorable pharmacokinetic and pharmacodynamic than clonidine [47], might be an interesting option for neuraxial anesthesia and analgesia [48]. Administered as an adjuvant, the synergistic analgesic effect of neuraxial DEX might be contributed to its high selective affinity to the spinal α_2_AR that is approximately 8∼10 times higher than that of clonidine [47]. There is no such study comparing the dose equivalence and peri-operative related cost between DEX and clonidine, but previous studies have stated that the dose of clonidine is 1.5∼4 times greater than DEX when it is delivered *via* epidural route [37], [49]. Since the analgesic effect of DEX was mainly mediated *via* the α_2_AR [50], the cardio-respiratory adverse events *via* α_1_AR might be minimized. There were several included studies reporting that neuraxial DEX was associated with a lower inspired inhalation anesthetic concentration [31], [35], [36] and bispectral index (BIS) [30] compared with placebo group, indicating that a synergism between neuraxial DEX and LAs yielded to anesthetic sparing and improved anesthesia.

The pooled results from our meta-analysis showed that adjunct neuraxial DEX was associated with significantly lower pain intensity within 24 hours postoperatively compared with placebo group. The average decrease in pain intensity was approximate 1.3 on a VRS, VAS, FLACC, or CHIPPS scale, indicating a mild to moderate postoperative pain relief. The previous meta-analyses [18], [19] didn’t pool this part of results because of the limited number of included studies and significant clinical heterogeneity (DEX *via* neuraxial *vs.* peripheral or intravenous *vs.* intrathecal). We also demonstrated that the duration of postoperative analgesia in neuraxial DEX group was prolonged by approximate 7 hours, which was longer than previous studies (approximate 4 and 5 hours, respectively) [18], [19]. This discrepancy was derived from the clinical heterogeneity. Our further subgroup analysis revealed the similarly prolonged duration of postoperative analgesia in intrathecal DEX group (approximate 4.2 hours). Caudal DEX tended to prolong more analgesic duration compared with epidural or intrathecal DEX (10 *vs.* 2 *vs.* 4 hours), however, this pooled results might be weakened by clinical heterogeneity that caudal anesthesia in 4 included studies was all performed in children.

The pooled results from our meta-analysis showed that adjunct neuraxial DEX was associated with a significantly quick onset of sensory and motor block, and prolonged duration of sensory block compared with placebo group, which was similar to previous reports [18], [19]. Subgroup analyses revealed that a clinical heterogeneity, such as the different routes, doses of DEX, might influence the results. Although most of the subgroup results didn’t reach the statistically significant difference compared with the pooled ones, the prolonged duration of block time might be considered clinical difference (e.g. an average prolongation of duration of sensory block: approximate 43 minutes for ≤ 5 μg DEX *vs.* 102 minutes for > 5 μg DEX; an average prolongation of duration of motor block: approximate 90 minutes for intrathecal DEX *vs.* 120 minutes for intrathecal DEX *vs.* 8 minutes for caudal DEX).

The pooled results from our meta-analysis showed that adjunct neuraxial DEX was associated with a significant change in intra-operative hemodynamic compared with placebo group. However, an increase of approximate 1.4 bpm HR and decrease of 2 mmHg MAP were considered as no clinical significance. Eight trials [28], [29], [31]–[33], recorded the intra-operative ephedrine or atropine consumption, and no group difference was detected between neuraxial DEX and placebo group, suggesting an overall stable hemodynamic and that these changes were easily reversed.

The pooled results from our meta-analysis showed that adjunct neuraxial DEX was associated with a significantly higher incidence of bradycardia (NNH = 14) compared with placebo group, which was in agreement with previous reports [18], [19]. No evidence showed any increased risk of other adverse events, such as hypotension, nausea and vomiting. Six trials [28], [32], [33], [39], [42], [43] reported that no patient suffered from neurological impairment within 1 to 2 weeks follow-up.

However, our results might be weakened by several limitations. First, there were high heterogeneity in 2 primary outcomes (postoperative pain intensity and analgesia duration) and 4 secondary outcomes, since we pooled different route and dose of neuraxial DEX, different type of anesthesia, surgical procedural, and LAs, different postoperative time period, and different age and gender together in our analyses. Although a series of subgroup analyses and meta-regression were performed to identify the potential clinical and methodological heterogeneity, we failed to consolidate any cause to the significant heterogeneity. Thus, we used random effect model to modify the potential influence of heterogeneity on the result validity with wide 95% CI. Second, the limited number of included studies with varied clinical heterogeneity did not allow us to perform a detailed meta-regression including all possible predictors. Third, one primary outcome (postoperative pain intensity) might be influenced by publication bias indicated by Begg’s funnel plot and Egger’s test, since positive results are always more frequently published than the negative ones. A sensitivity analysis by excluding studies with low quality or high risk of bias revealed that the model and statistical assumptions did not influence our pooled results [51]. Fourth, six included studies with low Jadad scores [32]–[34], [36], [37], and 1 study with high risk of attrition bias [38] might influence our pooled results. Finally, although we have confirmed the favorable safety profile of neuraxial DEX in short-term, long-term outcomes concerning potential neurotoxicity and delayed neurological impairments are lacking.

## Conclusion

Our evidence demonstrated that neuraxial DEX is a favorable LA adjuvant with decreased postoperative pain intensity, prolonged analgesic duration and improved neuraxial anesthesia. The greatest concern is bradycardia. Since DEX has not been approved in most countries for neuraxial use yet, urge cautions regarding the use of neuraxial DEX are highlighted in medical practice. Further trials with strict design and focusing on long-term outcomes are warranted.

## Supporting Information

Figure S1 **Sensitivity analysis.** A: postoperative pain intensity, B: postoperative analgesic duration, C: bradycardia, and D: hypotension.
(TIF)

Figure S2 **Begg’s funnel plot.** A: postoperative pain intensity, B: postoperative analgesic duration, C: bradycardia, and D: hypotension.
(TIF)

Table S1 **Main study characteristics.**
(DOC)

Table S2 **Risk of bias for each included study.**
(DOC)

Table S3 **Egger’s test of primary outcomes.**
(DOC)

Checklist S1 **PRISMA checklist.**
(DOC)
