# Supplementary figures and images for: Does Dexmedetomidine as a Neuraxial Adjuvant Facilitate Better Anesthesia and Analgesia? A Systematic Review and Meta-Analysis
Source: PLoS One. 2014 Mar 26;9(3):e93114. doi: 10.1371/journal.pone.0093114 (PMC3966844; doi:10.1371/journal.pone.0093114)

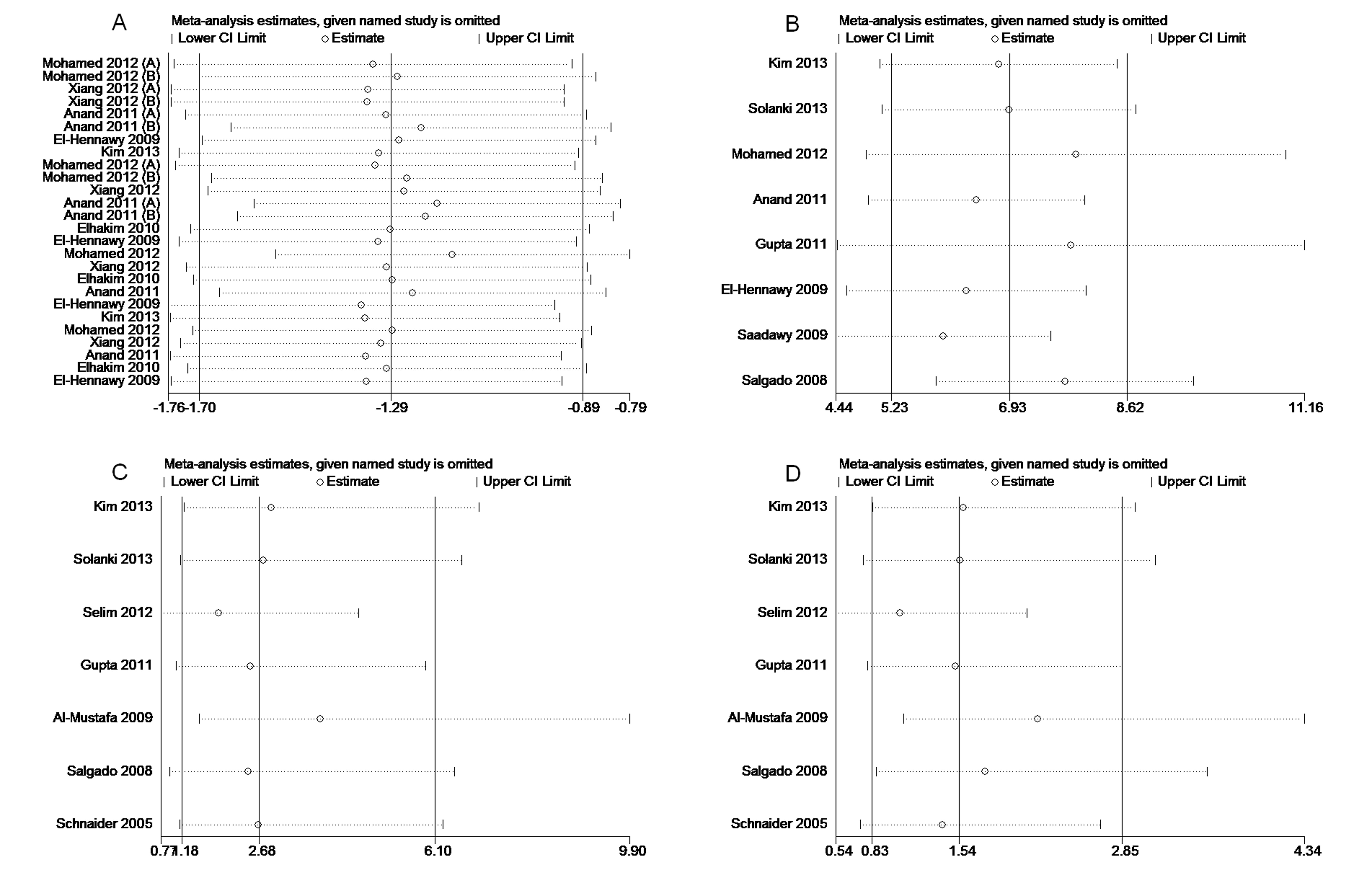

Supplement: Figure S1 — Sensitivity analysis. A: postoperative pain intensity, B: postoperative analgesic duration, C: bradycardia, and D: hypotension. (TIF) [file pone.0093114.s001.tif]

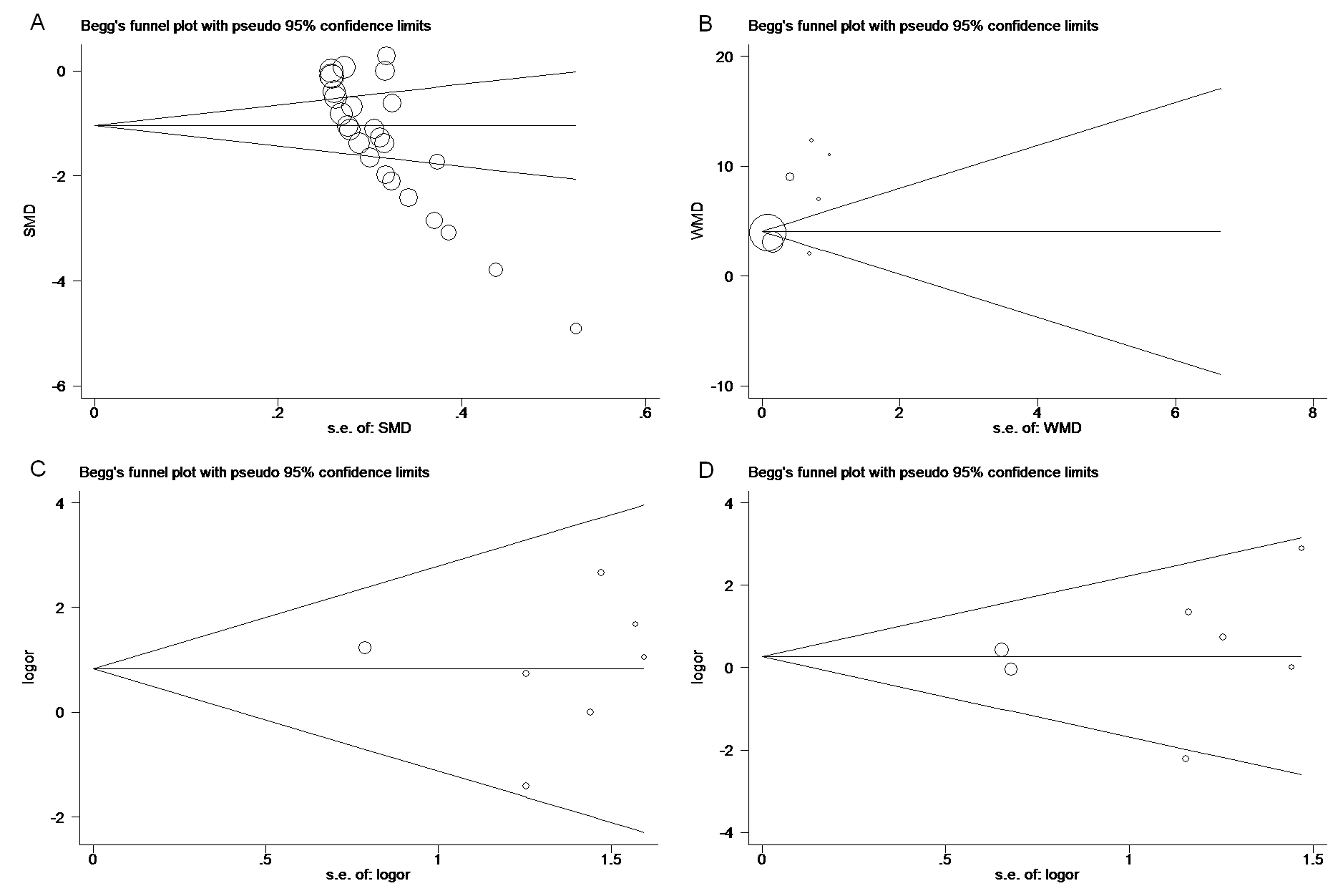

Supplement: Figure S2 — Begg’s funnel plot. A: postoperative pain intensity, B: postoperative analgesic duration, C: bradycardia, and D: hypotension. (TIF) [file pone.0093114.s002.tif]
