# Supplementary material for: Does Dexmedetomidine as a Neuraxial Adjuvant Facilitate Better Anesthesia and Analgesia? A Systematic Review and Meta-Analysis
Source: PLoS One. 2014 Mar 26;9(3):e93114. doi: 10.1371/journal.pone.0093114 (PMC3966844; doi:10.1371/journal.pone.0093114)
Supplement: Table S1 — Main study characteristics. (DOC) [file pone.0093114.s003.doc]

**Table S1.** Main Study Characteristics

| **Source**  **(country)** | **Patients** | | | **Intervention** | | |
| --- | --- | --- | --- | --- | --- | --- |
|  | **Total numbers** | **ASA I~II/%** | **Age, mean (range) y** | **DEX group** | **Placebo group** | **Multiple intervention** |
| **DEX *via* epidural route** | | | | | | |
| Selim, et al.  2012 (Egypt) | 130 | 130/130 (100) | 24  (17-31) | 0.25% Bupi 12 ml (30 mg) + 1 μg/kg DEX in NS 5 ml | 0.25% Bupi 12 ml (30 mg) + NS 5 ml | 0.25% Bupi 12 ml (30 mg) + 1 μg/kg DEX + 1 μg/kg Fen in NS |
| Elhakim, et al.  2010 (Egypt) | 50 | 26/50 (52) | 51  (40-60) | 0.5% Bupi 30-40 mg + 1 μg/kg DEX in NS^*^ | 0.5% Bupi 30-40 mg in NS^*^ | — |
| Salgado, et al.  2008 (Spain) | 40 | 40/40 (100) | 45.4  (18-60) | 0.75% Ropi 20ml (150 mg) + 1 μg/kg DEX in NS 1 ml | 0.75% Ropi 20 ml (150 mg) + NS 1 ml | — |
| Schnaider, et al.  2005 (Brazil) | 70 | 70/70 (100) | 39.5  (18-50) | 0.75% Ropi 20 ml (150 mg) + 2 μg/kg DEX in NS 1 ml^*^ | 0.75% Ropi 20 ml (150 mg) + NS 1 ml^*^ | 0.75% Ropi 20 ml (150 mg) + DEX 2 μg/kg + 0.5 mg/kg KTM in NS^*^ |
|  |  |  |  |  |  | 0.75% Ropi 20 ml (150 mg) + 150 μg clonidine in NS^*^ |
| **DEX *via* intrathecal route** | | | | | | |
| Kim, et al.  2013 (Korea) | 54 | NA | 67.7  (NA) | 0.5% hyperbaric Bupi 6 mg + 3 μg DEX in NS | 0.5% hyperbaric Bupi 6 mg + NS | — |
| Solanki, et al.  2013 (India) | 94 | 94/94 (100) | 33.3  (18-65) | 0.5% hyperbaric Bupi 15 mg + 5 μg DEX in NS 0.5 ml | 0.5% hyperbaric Bupi 15 mg + NS 0.5 ml | 0.5% hyperbaric Bupi 15 mg + 50 μg clonidine in NS |
| Mohamed, et al.  2012 (Egypt) | 90 | 90/90 (100) | 44.3  (25-55) | 0.5% hyperbaric Bupi 2 ml (10 mg) + 5 μg DEX in NS 1 ml^*^ | 0.5% Bupi 2 ml (10 mg) + NS 1 ml^*^ | 0.5% Bupi 2 ml (10 mg) + 5 μg DEX + 25 μg Fen in NS^*^ |
| Eid, et al.  2011 (Egypt) | 48 | 48/48 (100) | 29  (NA) | 3 ml hyperbaric Bupi 15 mg + 10 μg DEX in NS 0.5 ml | 3 ml hyperbaric Bupi 15 mg + NS 0.5 ml | — |
|  |  |  |  | 3 ml hyperbaric Bupi 15 mg + 15 μg DEX in NS 0.5 ml |  |  |
| Gupta, et al.  2011 (India) | 60 | 60/60 (100) | 40.8  (18-50) | 0.75% isobaric Bupi 3 ml (22.5 mg) + 5 μg DEX in NS 0.5 ml | 0.75% isobaric Bupi 3 ml (22.5 mg) + NS 0.5 ml | — |
| Shukla, et al.  2011 (India) | 90 | 90/90 (100) | NA  (18-45) | hyperbaric Bupi 15 mg + 10 μg DEX in NS 0.1 ml | hyperbaric Bupi 15 mg + NS 0.1 ml | hyperbaric Bupi 15 mg + 10 μg DEX + 50 mg Mg in NS |
| Al-Mustafa, et al.  2009 (Jordan) | 66 | 57/64 (89) | 64.4  (53-76) | 0.5% isobaric Bupi 2.5 ml (12.5 mg) + 5 μg DEX in NS 0.5 ml | 0.5% isobaric Bupi 2.5 ml (12.5 mg) + NS 0.5 ml | — |
|  |  |  |  | 0.5% isobaric Bupi 2.5 ml (12.5 mg) + 10 μg DEX in NS 0.5 ml |  |  |
| Kanazi, et al.  2006 (Lebanon) | 60 | 41/51 (80.4) | 69.7  (60-80) | hyperbaric Bupi 12 mg + 3 μg DEX in NS | hyperbaric Bupi 12 mg + NS | hyperbaric Bupi 12 mg + 3 μg DEX + 30 μg clonidine in NS |
| **DEX *via* caudal route** | | | | | | |
| Xiang, et al.  2012 (China) | 60 | 60/60 (100) | 42  (12-72) m | 0.25% Bupi 1 ml/kg + 1 μg/kg DEX in NS 1 ml | 0.25% Bupi 1 ml/kg + NS 1 ml | — |
| Anand, et al.  2011 (India) | 60 | 60/60 (100) | 42.7 m  (6 m-6 y) | 0.25% Ropi 1 ml/kg + 2 μg/kg DEX in NS 0.5 ml^*^ | 0.25% Ropi 1 ml/kg + NS 0.5 ml^*^ | — |
| El-Hennawy, et al.  2009 (Egypt) | 60 | 60/60 (100) | NA  (6 m-6 y) | 0.25% Bupi 1 ml/kg + 2 μg/kg DEX in NS 1 ml^*^ | 0.25% Bupi 1 ml/kg + NS 1 ml^*^ | 0.25% Bupi 1 ml/kg + 2 μg/kg DEX + 2 μg/kg clonidine in NS^*^ |
| Saadawy, et al.  2008 (Egypt) | 60 | 60/60 (100) | 31.3 m  (1-6 y) | 0.25% Bupi 1 ml/kg + 1 μg/kg DEX in NS | 0.25% Bupi 1 ml/kg in NS | — |

**Table S1.** Main Study Characteristics (Continued)

| **Study**  **(country)** | **Completed no./total(%)** | | **Neurological complication follow-ups** | **Outcome measures used for meta** | **Method Quality, jadad score** |
| --- | --- | --- | --- | --- | --- |
|  | **DEX group** | **Placebo group** |  |  |  |
| **DEX *via* epidural route** | | | | | |
| Selim, et al.  2012 (Egypt) | 44/50 (88) | 23/30 (76.7) | NA | adverse effect | 4 |
| Elhakim, et al.  2010 (Egypt) | 25/25 (100) | 25/25 (100) | NA | pain intensity | 4 |
| Salgado, et al.  2008 (Spain) | 19/20 (95) | 17/20 (85) | NA | analgesia, block characteristics, adverse events | 3 |
| Schnaider, et al.  2005 (Brazil) | 20/20 (100) | 10/10 (100) | NA | adverse events | 3 |
| **DEX *via* intrathecal route** | | | | | |
| Kim, et al.  2013 (Korea) | 27/27 (100) | 27/27 (100) | 1 wks following discharge | pain, block characteristics, hemodynamic, additional analgesic, adverse events | 5 |
| Solanki, et al.  2013 (India) | 30/32 (93.8) | 30/31 (96.8) | NA | block characteristics, additional analgesic, adverse events | 5 |
| Mohamed, et al.  2012 (Egypt) | 30/30 (100) | 30/30 (100) | NA | pain intensity, hemodynamic, additional analgesic, adverse events | 3 |
| Eid, et al.  2011 (Egypt) | 15/16 (93.75) | 16/16 (100) | 2 wks following discharge | pain intensity, block characteristics, additional analgesic, adverse events | 5 |
|  | 16/16 (100) |  |  |  |  |
| Gupta, et al.  2011 (India) | 30/30 (100) | 30/30 (100) | 1 wks following discharge | block characteristics, additional analgesic, adverse events | 3 |
| Shukla, et al.  2011 (India) | 30/30 (100) | 30/30 (100) | 2 wks following discharge | block characteristics | 5 |
| Al-Mustafa, et al.  2009 (Jordan) | 21/22 (95.5) | 22/22 (100) | 2 wks following discharge | hemodynamic, block characteristics, adverse events | 5 |
|  | 21/22 (95.5) |  |  |  |  |
| Kanazi, et al.  2006 (Lebanon) | 16/20 (80) | 19/20 (95) | 2 wks following discharge | hemodynamic, block characteristics, adverse events | 3 |
| **DEX *via* caudal route** | | | | | |
| Xiang, et al.  2012 (China) | 30/30 (100) | 30/30 (100) | NA | pain intensity, additional analgesic, sedation, adverse events | 3 |
| Anand, et al.  2011 (India) | 30/30 (100) | 30/30 (100) | NA | pain intensity, analgesia, hemodynamic | 4 |
| El-Hennawy, et al.  2009 (Egypt) | 20/20 (100) | 20/20 (100) | NA | pain intensity, analgesia, hemodynamic, additional analgesic, adverse events | 5 |
| Saadawy, et al.  2008 (Egypt) | 30/30 (100) | 30/30 (100) | NA | block characteristics, additional analgesic, hemodynamic, adverse events | 5 |
| * general anesthesia was conducted in these studies  **Abbreviations:** **ASA**, American Society of Anesthesiologist; **Bupi**, bupivocaine; **DEX**, dexmedetomidines; **Fen**, fentanyl; **KTM**, ketamin; Mg, magnesium; **NA**, not assessed; **NS**, normal saline; **Ropi**, ropivacaine | | | | | |
