# Supplementary material for: Does Dexmedetomidine as a Neuraxial Adjuvant Facilitate Better Anesthesia and Analgesia? A Systematic Review and Meta-Analysis
Source: PLoS One. 2014 Mar 26;9(3):e93114. doi: 10.1371/journal.pone.0093114 (PMC3966844; doi:10.1371/journal.pone.0093114)
Supplement: Table S2 — Risk of bias for each included study. (DOC) [file pone.0093114.s004.doc]

**Table S2.** Risk of bias for each included study

| **Study** | **Sequence generation (selection bias)** | **Allocation concealment (selection bias)** | **Blinding of participants and personnel (performance bias)** | **Blinding of outcome assessment (detection bias)** | **Incomplete outcome data (attrition bias)** | **Selective reporting (reporting bias)** | **Other bias** |
| --- | --- | --- | --- | --- | --- | --- | --- |
| Selim, et al. 2012 | Unknown | Low risk | Low risk | Low risk | High risk | Unknown | Unknown |
| Elhakim, et al. 2010 | Unknown | Low risk | Low risk | Low risk | Low risk | Unknown | Low risk |
| Salgado, et al. 2008 | Unknown | Unknown | Low risk | Low risk | Low risk | Unknown | Unknown |
| Schnaider, et al. 2005 | Unknown | Unknown | Low risk | Low risk | Low risk | Unknown | Unknown |
| Kim, et al. 2013 | Low risk | Low risk | Low risk | Low risk | Low risk | Unknown | Low risk |
| Solanki, et al. 2013 | Low risk | Low risk | Low risk | Low risk | Low risk | Unknown | Low risk |
| Mohamed, et al. 2012 | Unknown | Unknown | Low risk | Low risk | Low risk | Unknown | Unknown |
| Eid, et al. 2011 | Low risk | Low risk | Low risk | Low risk | Low risk | Unknown | Low risk |
| Gupta, et al. 2011 | Unknown | Unknown | Low risk | Low risk | Low risk | Unknown | Unknown |
| Shukla, et al. 2011 | Low risk | Low risk | Low risk | Low risk | Low risk | Unknown | Low risk |
| Al-Mustafa, et al. 2009 | Low risk | Low risk | Low risk | Low risk | Low risk | Unknown | Low risk |
| Kanazi, et al. 2006 | Unknown | Unknown | Low risk | Low risk | Low risk | Unknown | Unknown |
| Xiang, et al. 2012 | Unknown | Unknown | Low risk | Low risk | Low risk | Unknown | Unknown |
| Anand, et al. 2011 | Low risk | Unknown | Low risk | Low risk | Low risk | Unknown | Low risk |
| El-Hennawy, et al. 2009 | Low risk | Low risk | Low risk | Low risk | Low risk | Unknown | Low risk |
| Saadawy, et al. 2008 | Low risk | Low risk | Low risk | Low risk | Low risk | Unknown | Low risk |
