# Supplementary material for: Does Dexmedetomidine as a Neuraxial Adjuvant Facilitate Better Anesthesia and Analgesia? A Systematic Review and Meta-Analysis
Source: PLoS One. 2014 Mar 26;9(3):e93114. doi: 10.1371/journal.pone.0093114 (PMC3966844; doi:10.1371/journal.pone.0093114)
Supplement: Table S3 — Egger’s test of primary outcomes. (DOC) [file pone.0093114.s005.doc]

**Table S3.** Egger’s test of primary outcomes

| **Primary outcomes** | **Slope** | | | | | **Bias** | | | | |
| --- | --- | --- | --- | --- | --- | --- | --- | --- | --- | --- |
|  | **Coef.** | **Std. Err.** | **t** | **P > \|t\|** | **95% CI** | **Coef.** | **Std. Err.** | **t** | **P > \|t\|** | **95% CI** |
| Postoperative pain intensity | 4.43 | 0.76 | 5.80 | 0.00 | 2.85, 6.00 | -18.21 | 2.51 | -7.25 | 0.00 | -23.40, -13.03 |
| Postoperative analgesia duration | 3.21 | 0.66 | 4.87 | 0.003 | 1.60, 4.83 | 5.84 | 3.05 | 1.92 | 0.10 | -1.62, 13.29 |
| Bradycardia | 0.96 | 1.93 | 0.50 | 0.64 | -4.01, 5.92 | -0.10 | 1.57 | -0.07 | 0.95 | -4.14, 3.93 |
| Hypotension | -0.39 | 1.50 | -0.26 | 0.80 | -4.24, 3.45 | 0.73 | 1.56 | 0.47 | 0.66 | -3.28, 4.73 |
